# Identification and utilization of inter-species conserved (ISC) probesets on Affymetrix human GeneChip^® ^platforms for the optimization of the assessment of expression patterns in non human primate (NHP) samples

**DOI:** 10.1186/1471-2105-5-165

**Published:** 2004-10-26

**Authors:** Zhining Wang, Mark G Lewis, Martin E Nau, Alma Arnold, Maryanne T Vahey

**Affiliations:** 1 Henry M Jackson Foundation for the Advancement of Military Medicine, Rockville, Maryland 20850, USA; 2 Division of Retrovirology, Walter Reed Army Institute of Research, Washington, D. C. 20850, USA; 3 Bioqual, Rockville, MD 20850, USA

## Abstract

**Background:**

While researchers have utilized versions of the Affymetrix human GeneChip^® ^for the assessment of expression patterns in non human primate (NHP) samples, there has been no comprehensive sequence analysis study undertaken to demonstrate that the probe sequences designed to detect human transcripts are reliably hybridizing with their orthologs in NHP. By aligning probe sequences with expressed sequence tags (ESTs) in NHP, inter-species conserved (ISC) probesets, which have two or more probes complementary to ESTs in NHP, were identified on human GeneChip^® ^platforms. The utility of human GeneChips^® ^for the assessment of NHP expression patterns can be effectively evaluated by analyzing the hybridization behaviour of ISC probesets. Appropriate normalization methods were identified that further improve the reliability of human GeneChips^® ^for interspecies (human vs NHP) comparisons.

**Results:**

ISC probesets in each of the seven Affymetrix GeneChip^® ^platforms (U133Plus2.0, U133A, U133B, U95Av2, U95B, Focus and HuGeneFL) were identified for both monkey and chimpanzee. Expression data was generated from peripheral blood mononuclear cells (PBMCs) of 12 human and 8 monkey (Indian origin *Rhesus macaque*) samples using the Focus GeneChip^®^. Analysis of both qualitative detection calls and quantitative signal intensities showed that intra-species reproducibility (human vs. human or monkey vs. monkey) was much higher than interspecies reproducibility (human vs. monkey). ISC probesets exhibited higher interspecies reproducibility than the overall expressed probesets. Importantly, appropriate normalization methods could be leveraged to greatly improve interspecies correlations. The correlation coefficients between human (average of 12 samples) and monkey (average of 8 Rhesus macaque samples) are 0.725, 0.821 and 0.893 for MAS5.0 (Microarray Suite version 5.0), dChip and RMA (Robust Multi-chip Average) normalization method, respectively.

**Conclusion:**

It is feasible to use Affymetrix human GeneChip^® ^platforms to assess the expression profiles of NHP for intra-species studies. Caution must be taken for interspecies studies since unsuitable probesets will result in spurious differentially regulated genes between human and NHP. RMA normalization method and ISC probesets are recommended for interspecies studies.

## Background

Microarray studies on non human primates (NHP) have been used to address viral pathogenesis [1,2], neurological disorders [3], development [4] and phylogenetic studies [5-7]. Due to the lack of species-specific microarray platforms for NHP, researchers have used GeneChip^® ^platforms built using human sequence information. An underlying assumption in such studies is that transcripts of humans and NHP are highly conserved, and probe sequences designed to detect human genes will detect their orthologs in NHP samples. It is estimated that chimpanzees (*Pan troglodytes*) and humans shared 98.77 % DNA similarity [8]. While this statistic is widely quoted and believed, Britten [9] reported that the divergence between humans and chimpanzees to be about 5%. Anzai and colleagues [10] compared the chimpanzee MHC region (1,750,601 bp) with the human HLA region (1,870,955 bp), and concluded that the similarity drops to 86.7% if insertions and deletions were taken into account. All these analyses are based on genomic DNA sequences; however, for microarray studies on the transcriptome, the similarity of RNA transcripts is the primary concern. A single gene does not necessarily generate a single transcript. Splicing variants are very common in the human [11,12], and humans and NHPs may use different splicing strategies in some genes. Therefore, it is necessary to re-assess the reliability of human GeneChips^® ^for NHP expression analysis.

Few published studies employing human GeneChip^® ^platforms for NHP expression profiling have robustly addressed the quantitative aspects of cross platform performance. Vahey and colleagues [1] used the HuGeneFL GeneChip^® ^and demonstrated that there was no significant difference in the dynamic range of the raw fluorescence distribution for equivalent amounts of human cRNA and macaque cRNA hybridized to the chip. Chismar and colleagues [13] used the U95Av2 GeneChip^® ^platform and compared the expression patterns of humans with that of the rhesus macaque. They concluded that the percentage of 'present' calls observed in the transcriptome of macaque brain is lower than that of human brain, and that this is especially true for genes with lower signal intensity. Caceres and colleagues [5] used the HG-U95Av2 arrays to identify upregulated genes in the human cortex compared with those of the NHPs. Since sequence divergence could lead to an underestimation of expression levels in NHPs, they excluded 4572 probes that exhibited different hybridization behaviour between two sets of samples in order to reduce false positives. However, this analysis is solely based on probe signal intensities. A more robust way to assess the utility of human GeneChip^® ^platforms for the study of expression profiles in NHP is to employ a sequence analysis approach.

In this study, we address the power of human GeneChip^® ^platforms to assess expression patterns in NHP samples by: a) identifying ISC probesets based on sequence analysis; b) assessing intra (within NHP species)- and interspecies (between NHP and human samples) reproducibility of GeneChip^® ^data; and c) applying appropriate normalization methods to improve interspecies reproducibility.

## Results and discussion

### Identification of ISC probesets

**Figure 1:**
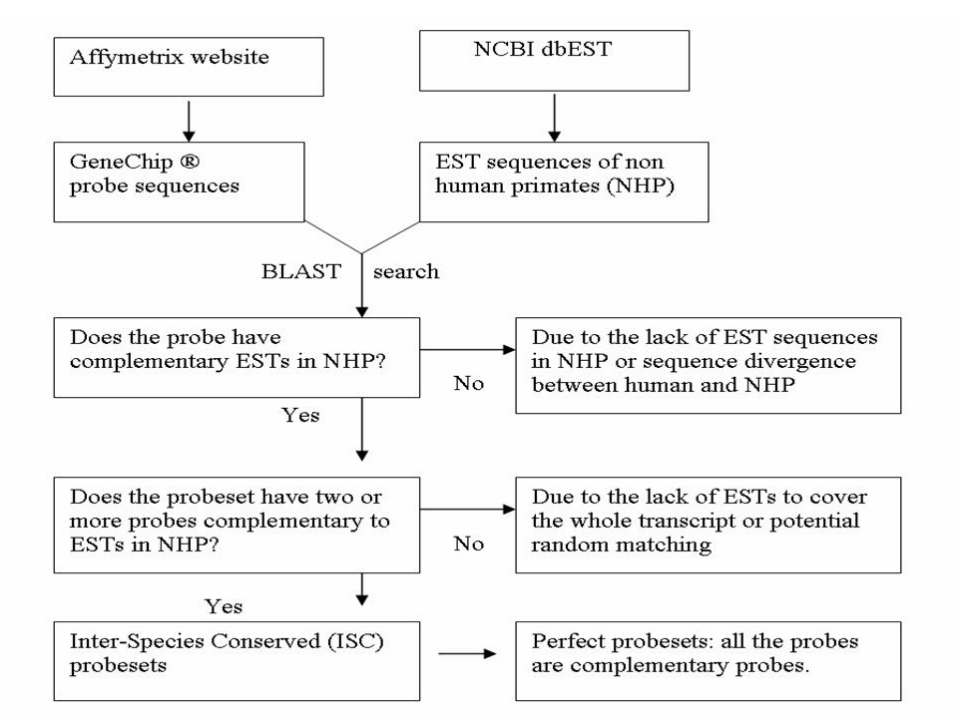
**Algorithm for identifying ISC probesets in Affymetrix Human GeneChip^® ^platforms. **In the Affymetrix platform, a probe is a 25-mer oligo. A set of 11–20 probes forms a probeset. An ISC probeset is defined as having at least two probes that are complementary to ESTs in NHP. A perfect probeset is the one that all of its probes are complementary to ESTs in NHP.

When a probe sequence on the human GeneChip^® ^hybridizes with the transcriptome of a NHP, there are three possible outcomes: 1) it hybridizes with the ortholog of the NHP; 2) it cross-hybridizes with a non-ortholog transcript, or 3) it fails to hybridize due to sequence divergence. In Affymetrix GeneChip^® ^system, a probeset is composed of 11–20 probes and each probe is a 25-mer oligo. We identified probes on the human GeneChip that are complementary to ESTs in NHP postulating that these probes would hybridize most optimally with the transcripts of NHP. We defined a probeset as an ISC probeset if it had at least two complementary probes. The rationale used to define the criterion that defines an ISC probeset is described in the methods section. The procedure used to generate ISC probesets is shown in Figure 1 and described in methods section. ISC probesets in each of the seven Affymetrix human GeneChip^® ^platforms (U133Plus2.0, U133A, U133B, U95Av2, U95B, Focus and HuGeneFL) were generated for both monkey and chimpanzee. Detailed information about each ISC probeset such as probe sequence, GenBank accession and the position and degree of matching is provided in the supplemental materials (Additional File 1,Additional File 2,Additional File 3,Additional File 4). Table 1 displays a summary of the statistical characteristics of ISC probesets. Not surprisingly, there were more ISC probesets for monkey (*Macaca mulatta*) than for chimpanzee (*Pan troglodytes*). This is not because monkey EST sequences are more similar to human sequences than chimpanzee EST sequences, but because we have a much greater amount of EST sequences available for monkey. At the time of writing this manuscript, there were 33,474 monkey ESTs available, while there were only 6,943 ESTs available for chimpanzee. As the number of defined ESTs will increase in the future, additional ISC probesets could be identified for both monkey and chimpanzee using this method.

**Table 1:** The number of ISC probesets in various human GeneChip^® ^platforms

Human GeneChip^® ^platforms	Probes / probeset	Total number of probesets (genes*)	The number of ISC probesets (genes)	
			
			Monkey	Chimpanzee
HG-FL	20	7129 (5435)	1036 (891)	422 (362)
HG-Focus	11	8793 (8466)	1179 (1136)	523 (511)
HG-U95Av2	16	12625 (9203)	1505 (1267)	586 (511)
HG-U95B	16	12620 (9948)	561 (497)	256 (236)
HG-U133A	11	22283 (13624)	2676 (1991)	1102 (861)
HG-U133B	11	22646 (16119)	886 (773)	406 (363)
HG-U133 Plus2.0	11	54675 (29963)	3636 (2704)	1529 (1190)

* Refer to the number of unique UniGene clusters.

It is not uncommon, especially in the U133Plus2.0 platform, that multiple probesets target the same gene. For example, in the U133A and the U133 Plus 2.0 GeneChip^®^s, there are three probesets (217028_at, 211919_s_at and 209201_x_at) that target the gene CXCR4 at different positions in its transcript. In order to address this redundancy issue, we converted the number of probesets into the number of unique UniGene clusters based on the GeneChip^® ^annotation file provided by Affymetrix Website [18]. While a UniGene cluster does not necessarily correspond to a unique gene, it is a reasonable way to assess probeset redundancy. As shown in Table 1, the Focus GeneChip^® ^and the U133Plus2.0 GeneChip^® ^have the lowest and highest frequency of redundant probesets for a given gene, respectively.

**Figure 2:**
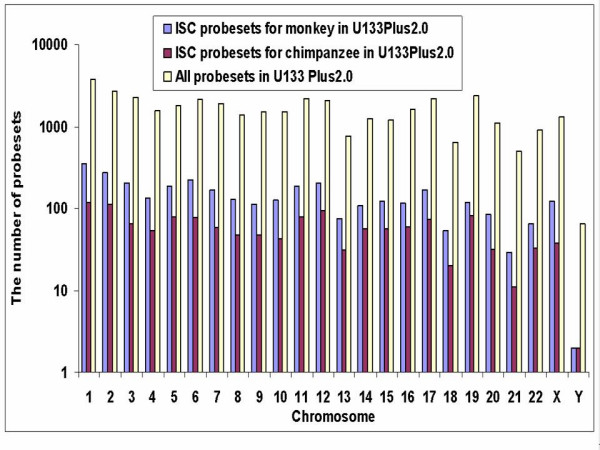
**Distribution of ISC probesets on human chromosomes. **The yellow bars represent the distribution of all probesets in GeneChip^® ^U133Plus2.0 platform. The blue and red bars represent the distribution of ISC probesets for monkey (*Macaca mulatta*) and chimpanzee (*Pan troglodytes*), respectively.

The U133Plus2.0 is the most current version of human GeneChip^® ^from Affymetrix and covers the human genome most extensively. Figure 2 displays the distribution of probesets on the human chromosomes. The yellow bars represent the distribution of all probesets on the U133Plus2.0 platform, and the blue and red bars represent the distribution of ISC probesets for monkey and chimpanzee, respectively. As shown in Figure 2, ISC probesets for both monkey and chimpanzee are distributed throughout the genome, from chromosome 1 to chromosome 22, including the two sex chromosomes X and Y. The percentage of ISC probesets on each chromosome is roughly proportional to that of the total probesets.

### Intra- and interspecies reproducibility of detection calls

**Figure 3:**
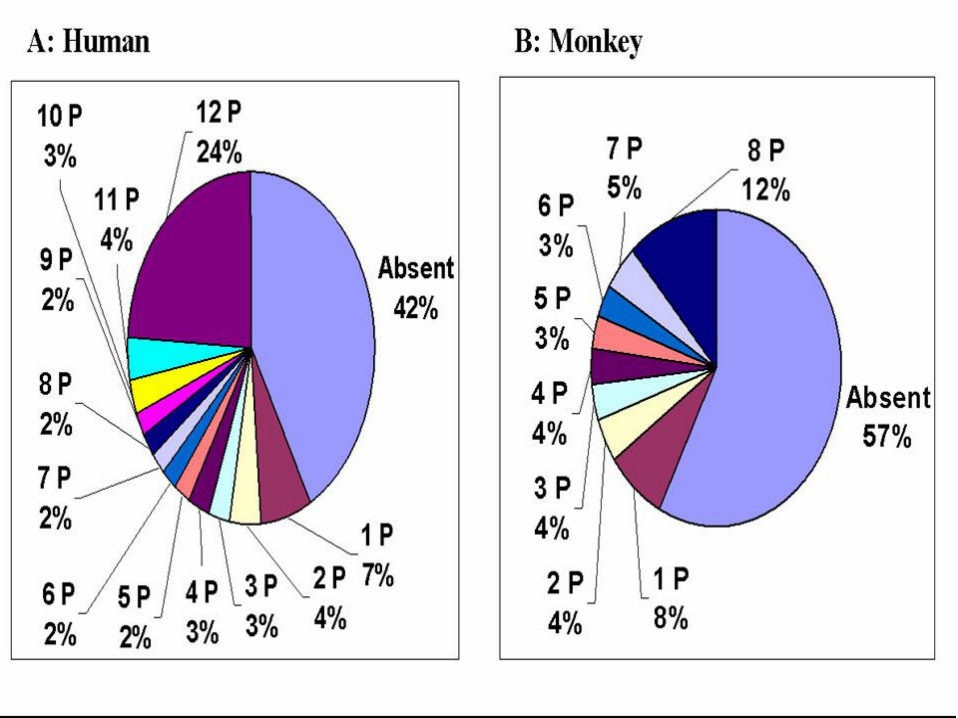
**Intra-species reproducibility of detection calls. A**: Reproducibility among human samples. **B: **Reproducibility among monkey (*Rhesus macaque*) samples. 1 P, 2 P ... 12 P represent 1, 2 ...12 present calls among all samples. Absent = no present calls in any sample. N = 12 human and 8 monkey samples.

The qualitative detection call (present / absent) output from MAS5.0 was the initial approach used to examine the reproducibility of GeneChip^® ^data observed in intra- and interspecies samples. The intra-species reproducibility is displayed in Figure 3A and 3B for human samples and monkey samples, respectively. As shown in Figure 3A, 66% of probesets showed 100% reproducibility across 12 human replicates, being either present in all samples (24%) or absent in all samples (42%). Similarly, among 8 monkey samples, 69% of probesets showed 100% reproducibility, being either present in all samples (12%) or absent in all samples (57%) (Figure 3B). Although the percentage of absent calls in monkey samples (57%) is higher than those in human samples (42%), the detection call itself is consistent across replicates. In other words, an absent call caused by sequence divergence will be reliably repeated across monkey samples. This result suggests that it is feasible to use the human GeneChip^® ^for NHP intra-species studies.

**Figure 4:**
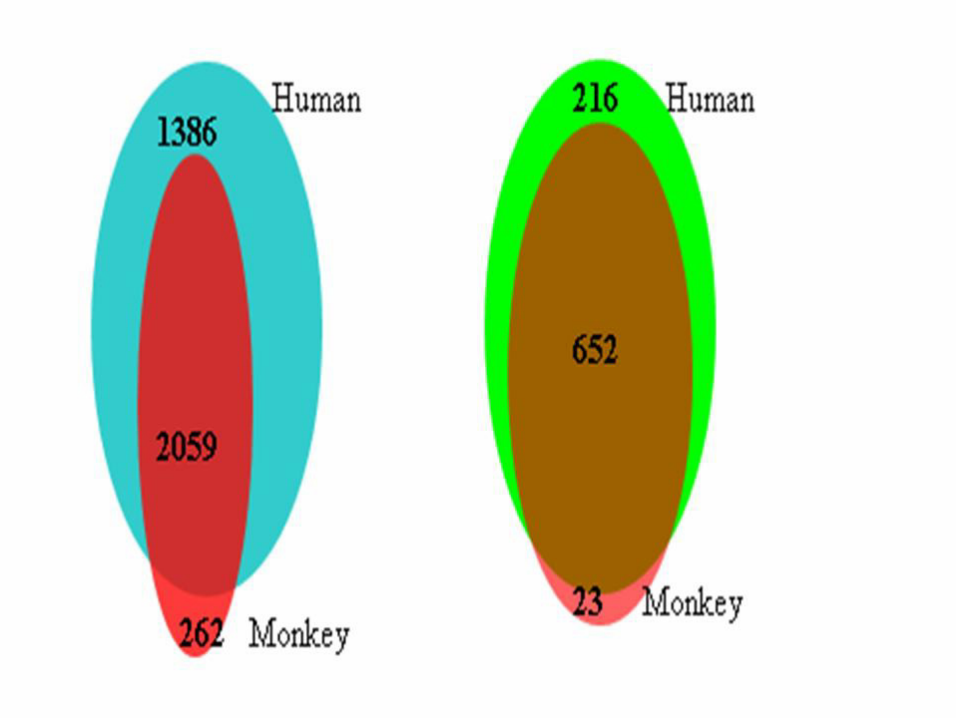
**Interspecies reproducibility of detection calls. A: **Venn diagram of the number of expressed probesets in human and monkey (*Rhesus macaque*) samples; **B: **Venn diagram of the number of expressed ISC probesets in human and monkey (*Rhesus macaque*) samples.

In contrast, if human GeneChip^® ^platforms are used to compare the expression pattern of humans with those of NHPs, care must be taken in the interpretation of data. If we consider a probeset as being expressed when 50% or more of replicates have present calls, then 3445 (2059+1386) and 2321 (2059+262) probesets are expressed in the PBMC fraction of humans and monkeys, respectively (Figure 4A). Approximately 40% (1386/3445) of probesets being detected in human PBMCs are not detected in the monkey. Due to the close evolutionary relationship between human and monkey, one would not expect that 40% of genes expressed in human PBMCs are not expressed in monkey PBMCs. This observation suggests that a subset of human probesets failed to properly hybridize with the orthologs of monkey. Based on expression data alone, however, it is difficult to distinguish a genuine absent call from a spurious absent call resulting from sequence divergence. ISC probesets can help to distinguish spurious from genuine absent calls. As shown in Figure 4B, of 868 ISC probesets that were detected in human PBMCs, only 216 (24.9%) are not detected in monkey PBMCs. The interspecies discordance is reduced significantly for ISC probesets (Fisher's exact test p < 2.2e-16). It is important to point out that ISC probesets will significantly reduce, but not eliminate interspecies discordance as it requires only a minimum of two complementary probes. It can be postulated that a perfect probeset in which all of its probes were complementary to ESTs of NHP would provide the ultimate reduction in discordance. However, the identification of such probesets is limited by currently available sequence information.

### Intra- and interspecies reproducibility of signal intensities

**Figure 5:**
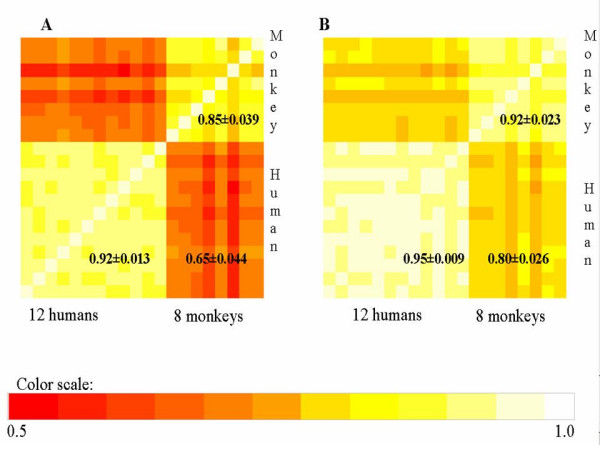
**Intra- and interspecies reproducibility of expression signal intensities. **Pair-wise correlation coefficients of 20 samples (12 human and 8 monkey (*Rhesus macaque*) samples) were calculated for expressed probesets (Figure 5A) and for expressed ISC probesets (Figure 5B). Correlation coefficients are visualized using colors of a heat spectrum (red=correlation coefficient of 0.5; white = correlation coefficient of 1.0). The graphs are symmetric along the diagonal lines. The diagonal line represents samples correlating with themselves, with a correlation coefficient of 1.0 (white). The means and standard deviations of correlation coefficients of human-human, monkey-monkey and human-monkey are shown in the bottom left, upper right and bottom right of each graph, respectively. **A**: Correlation coefficients calculated based on all expressed probesets. **B**: Correlation coefficients calculated based on expressed ISC probesets.

To assess the intra- and interspecies reproducibility of GeneChip^® ^signal intensities, a matrix that contains signal intensities of 3445 expressed probesets across 20 samples (12 human and 8 monkeys) was created. Probesets that are not expressed in human PBMCs were excluded in this analysis. Pair-wise correlation coefficients were calculated for all 20 samples (_20_C_2 _= 190 combinations in total). The correlation coefficients were visualized using heat spectrum graphs where colors ranging from red to white correspond to correlation coefficients of 0.5 to 1.0, respectively. In figure 5A, the cells in the diagonal line are all white as they represent samples correlating with themselves with a correlation coefficient of 1.0. The highest correlations were found among human replicates (lower left corner), followed by monkey replicates (upper right corner). The lowest correlations were found in interspecies comparisons (bottom right corner). The means and standard deviations of human-human, monkey-monkey and human-monkey correlation coefficients are 0.92 ± 0.013, 0.85 ± 0.039 and 0.65 ± 0.044, respectively. If the low correlation coefficients of human-monkey are caused by unsuitable probesets, then ISC probesets should have higher correlation coefficients. Figure 5B displayed the correlation coefficients of the same 20 samples as Figure 5A, but limited to ISC probesets. As shown in Fig 5B, the colors are much less red than those in Figure 5A, indicating higher correlation coefficients. The means and standard deviations of correlation coefficients of ISC probesets for human-human, monkey-monkey and human-monkey are 0.95 ± 0.0094, 0.92 ± 0.023 and 0.80 ± 0.026, respectively. The greatest improvement (0.65 to 0.80) in correlation coefficients are observed in the human-monkey comparison (Figure 5A and 5B) using the ISC probesets. This data suggests that a subset of problematic probesets interfered with interspecies comparison, and the ISC probesets could be used to improved interspecies reproducibility.

### The effect of normalization methods on interspecies reproducibility

**Figure 6:**
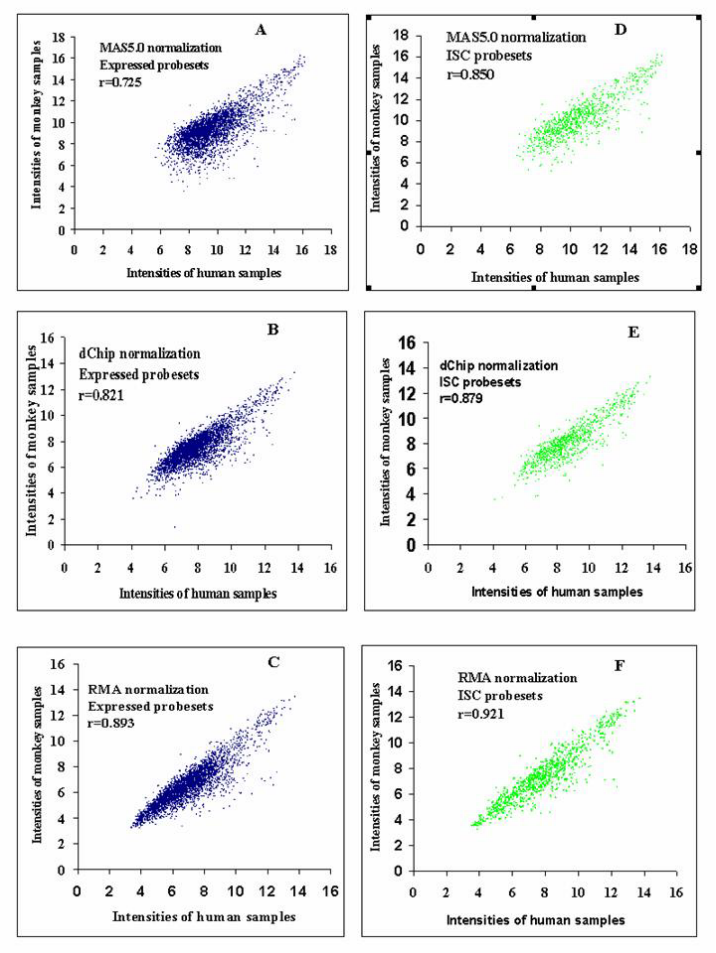
**The effect of normalization methods on interspecies reproducibility. **A, B and C: MAS5.0, dChip and RMA normalization for expressed probesets. D,E, and F: MAS5.0, dChip and RMA normalization for expressed ISC probesets. x-axes and y-axes are average expression intensities of 12 human samples and 8 monkey (*Rhesus macaque*) samples, respectively.

Different normalization methods have been shown to significantly affect GeneChip^® ^data variation [14-17]. We compared three different normalization methods: MAS5.0, RMA [14-16] and dChip [17], to evaluate the effect of normalization methods on interspecies reproducibility. Both RMA and dChip methods normalize GeneChip^® ^data at the probe level using a non-linear algorithm while MAS5.0 normalizes data at probeset level using linear scaling. Sequence divergence usually leads to one or very few probes in a probeset being problematic while the majority of probes in that probeset may still work reasonably well. If the variation generated from these problematic probes were normalized, the interspecies reproducibility should improve. Figure 6 showed the interspecies correlation coefficients using three different normalization methods. The average signal intensities of 8 monkey samples were given on the ordinate and that of 12 human samples on the abscissa. The RMA normalization method improved interspecies reproducibility the most for both expressed probesets and ISC probesets. As shown in the Figure 6A,6B,6C, correlation coefficients for expressed probesets using MAS5.0, dChip and RMA were 0.725, 0.821 and 0.893, respectively. Similarly, in Figure 6D,6E,6F, correlation coefficients for ISC probesets using MAS5.0, dChip and RMA were 0.850, 0.879 and 0.921, respectively. For the same normalization method, ISC probesets exhibited higher correlation coefficients than those of expressed probesets (horizontal comparison such as Figure 6A vs. Figure 6D). Use of the RMA normalization method in conjunction with the use of ISC probesets optimized the correlation coefficient between human and monkey. The resulting correlation coefficient of 0.92 is equivalent to the human-human correlation using the MAS5.0 normalization method (Figure 6F and Figure 5A).

## Conclusions

This paper presents a comprehensive analysis of probe sequences and GeneChip^® ^expression data as applied to the derivation of meaningful expression profile data from NHP. The utility of the human Affymetrix GeneChip^® ^for the assessment of expression profiles in NHP depends on the experimental design and on the approach to data normalization and analysis. Our observations suggest that: 1) it is feasible to use the human GeneChip^® ^in the evaluation of expression profiles of NHP samples for intra-species comparisons; 2) use of ISC probesets and RMA normalization are recommended for interspecies studies; and 3) with the increasing amount of ESTs of NHP, additional ISC probesets (and perfect probesets) will be identified in the near future.

## Methods

### Sequence data source

Affymetrix GeneChip probe sequences were downloaded from Affymetrix website [18]. The ESTs (Expressed Sequence Tags) of monkey (*Macaca mulatta*) and chimpanzee (*Pan troglodytes*) were downloaded from NCBI website [19].

### Identification of ISC probesets

Stand alone BALST program was downloaded from NCBI website [19]. Perl script was written to automatically run BLAST search between GeneChip^® ^probe sequences and monkey /chimpanzee EST sequences. The length of a probe sequence is always 25 nucleotides while the number of probes in a probeset varies from 11 to 20 depending on GeneChip^® ^platforms (see Table 1). A certain degree of mismatch between a probe sequence and ESTs is allowed. If a probe has at least 23 nucleotides complementary to at least one EST sequence, this probe is designated as a complementary probe. If a probeset has at least two complementary probes, we defined this probeset as an ISC probeset. If all probes of a probeset are complementary probes, this probeset is called a 'perfect' probeset. The rationale for the definition of ISC probesets is as follows: 1) since each probe is a 25-mer oligo, the probability of random matching of one probe is 4^-25 ^thus, the probability of random matching of two probes goes down to 4^-50^, being exponentially reduced; 2) in comparison with an RT-PCR experiment, the primer length is equivalent to our probe length, and two primers (one forward and one backward) usually generate a unique sequence in a whole genome; 3) a probe sequence on the Affymetrix GeneChip^® ^is a well designed sequence with a single probe hybridizing with a unique transcript in whole transcriptome; and 4) since the EST sequences in NHP are very limited so far, most of them do not cover whole transcript such that a false negative could be generated if we require all the probes in a probeset being complementary to known ESTs. In order to convert probeset IDs to UniGene IDs and map them onto chromosomes, probeset annotation files were downloaded from Affymetrix website [18]. No animals or human samples were used for the purpose of this analysis. Affymetrix datasets used in this analysis are from other approved ongoing projects in our lab. The procedure used to process these samples was previously published [1].

Briefly, peripheral blood from healthy human and NHP (Indian origin *Rhesus macaque*) was collected and peripheral blood mononuclear cells (PBMCs) were separated by Histopaque-Ficoll (Sigma) gradient centrifugation. RNA preparation, Hybridization, staining and scanning of the GeneChip^® ^was carried out as described by Vahey et al. [1]. Animal and human samples were handled identically throughout the process. All 20 samples (12 human and 8 rhesus macaques) were hybridized to Affymetrix's HG-Focus GeneChip^®^. Signal values and detection calls (present or absent) for all samples were determined by using MAS5.0 (Affymetrix Inc. Santa Clara, California). Signal values were scaled to the default target signal intensity of 500). A matrix of detection calls (present, absent and marginal) and a matrix of signal intensities for all samples across all probesets were constructed. A gene must exhibit 50% or more of 'present' calls in all samples to be considered 'expressed'. In this study, an expressed probeset in human is a probeset that has 6 or more present calls among 12 human samples. Similarly, an expressed probeset in monkey means there were 4 or more present calls among 8 monkey samples. The signal intensities output from MAS5.0 were log2 transformed. Model-based normalization was performed using dChip version 1.3 [17]. The output signal intensities were log2 transformed. RMA (Robust Multichip Average) normalization [14-16] was carried out using BioConductor package Affy_1.2.30 [20]. The *rma() *function in the package was used at its default setting, that is, 'RMA' background correction, 'quantile normalization', 'PM only model' and 'median polish summarization'. By default, the signal intensities were already log2 transformed.

Intra- and interspecies correlation coefficients of signal intensities were calculated by built in function '*cor*' in statistical package R version 1.9.0. [21]. Visualization of correlation coefficients matrix was done by the function '*image*'. The function '*heat.colors*' was used to create heat-spectrum (red to white) and set color scales between 0.5 (red) and 1.0 (white).

## Abbreviations

NHP: non human primate

EST: expressed sequence tag

ISC: inter-species conserved

RMA: robust multi-chip average

MAS5.0: Microarray suite version 5.0

## Authors' contributions

ZW developed the original hypotheses, performed the bioinformatics analyses to test them and drafted the manuscript. MV provided critical input on design and execution of the laboratory experiments and with ZW interpreted the data sets and revised the manuscript. ML conducted all aspects of the animal handling including the harvest of well characterized primary samples. MN and AA are technical staff who extracted the nucleic acid and performed the laboratory portions of the microarray experiments. All authors read and approved the final manuscript.

## Supplementary Material

Additional File 1 There are three worksheets in this file. Worksheet 1, 2 and 3 are ISC probesets for monkey (*Macaca mulatta*) in GeneChip^® ^platforms Plus2.0, U133A and U133B, respectively. Note: All the four additional files are multiple-sheets MS excel files. Within each worksheet of a file, the rows are ISC probesets, the columns are probeset IDs, positions of a probe (x and y coordinates in the chip), GenBank accessions of a matching EST, probe sequence, matched EST sequence and position, BLAST *e*- values and matching identities, in that order. Click here for file

Additional File 2 There are four worksheets in this file. Worksheet 1, 2, 3 and 4 are ISC probesets for monkey (*Macaca mulatta*) in GeneChip^® ^platforms Focus, FL, U95Av2 and U95B, respectively. Note: All the four additional files are multiple-sheets MS excel files. Within each worksheet of a file, the rows are ISC probesets, the columns are probeset IDs, positions of a probe (x and y coordinates in the chip), GenBank accessions of a matching EST, probe sequence, matched EST sequence and position, BLAST *e*- values and matching identities, in that order. Click here for file

Additional File 3 There are three worksheets in this file. Worksheet 1, 2 and 3 are ISC probesets for chimpanzee (*Pan troglodytes*) in GeneChip^® ^platforms Plus2.0, U133A and U133B, respectively. Note: All the four additional files are multiple-sheets MS excel files. Within each worksheet of a file, the rows are ISC probesets, the columns are probeset IDs, positions of a probe (x and y coordinates in the chip), GenBank accessions of a matching EST, probe sequence, matched EST sequence and position, BLAST *e*- values and matching identities, in that order. Click here for file

Additional File 4 There are four worksheets in this file. Worksheet 1, 2, 3 and 4 are ISC probesets for chimpanzee (*Pan troglodytes*) in GeneChip^® ^platforms Focus, FL, U95Av2 and U95B, respectively. Note: All the four additional files are multiple-sheets MS excel files. Within each worksheet of a file, the rows are ISC probesets, the columns are probeset IDs, positions of a probe (x and y coordinates in the chip), GenBank accessions of a matching EST, probe sequence, matched EST sequence and position, BLAST *e*- values and matching identities, in that order. Click here for file
